# Floral resource wastage: Most nectar produced by the mass‐flowering crop oilseed rape (*Brassica napus*) is uncollected by flower‐visiting insects

**DOI:** 10.1002/ece3.11453

**Published:** 2024-05-21

**Authors:** Ciaran Harris, Nicholas J. Balfour, Francis L. W. Ratnieks

**Affiliations:** ^1^ Laboratory of Apiculture and Social Insects (LASI), School of Life Sciences University of Sussex Brighton UK

**Keywords:** floral resources, mass‐flowering crops, plant‐pollinator interactions

## Abstract

Cultivation of the mass‐flowering crop oilseed rape (OSR), *Brassica napus*, can provide insects with super‐abundant nectar and pollen while in bloom. Several authors have suggested breeding cultivars to produce more abundant nectar and pollen to help mitigate insect decline. However, in Britain most, 95%, OSR blooms in spring (March–May), which has been suggested to be a period of nectar surplus and reduced exploitative competition. Therefore, a large proportion of floral resources produced by OSR during this period may be uncollected. Although there has been extensive work examining OSR nectar and pollen production, no study, to our knowledge, has measured this in relation to the demand by the flower‐visiting insects. Here we quantified the percentage of nectar produced by spring blooming OSR which was uncollected in four OSR fields per year over 2 years. This was achieved by measuring the nectar in both insect accessible and inaccessible (i.e. mesh‐covered) flowers. We also quantified uncollected pollen in flowers at the beginning and the end of anthesis using a haemocytometer. Most of the nectar (69%) and a fifth of pollen (19%) was uncollected in spring blooming OSR. Based on the estimates of nectar production and observed number of insects, nectar supply per insect was estimated at 2204 μL nectar insect^−1^ h^−1^, which exceeds potential collection rates by flower‐visiting insects. Given the majority of *B. napus* is spring blooming, breeding cultivars of OSR which produce more nectar, while not being detrimental to flower‐visiting insects, may be of little conservation benefit.

## Graphical abstract

Previous work has quantified the highly abundant nectar and pollen produced by OSR, and some authors have recommended breeding cultivars that produce more floral resources to help insects. We show that a high proportion of floral resources produced by spring blooming OSR are uncollected. Additionally, OSR fields produce nectar at a rate which far exceeds the rate which could be collected by active insects.

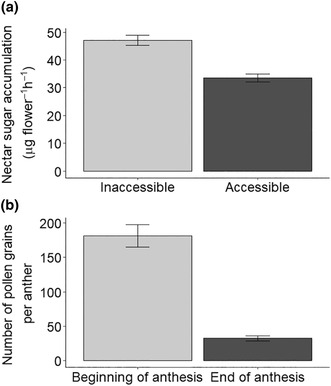

## 1 INTRODUCTION

Agricultural intensification and associated land use changes are major drivers of pollinator decline (Tscharntke et al., 2005). Examples include expansion of cultivated land in semi‐natural habitats (Blackstock et al., 1999; Ratcliffe, 1984), hedgerow removal (Petit et al., 2003), replacement of hay meadows with intensively managed pastures (Fitzpatrick et al., 2007; Green & Stowe, 1993), and increased fertiliser, herbicide, and pesticide applications (Ollerton et al., 2014; Potts et al., 2010). Many of these changes have reduced the abundance and diversity of flowers and increased competition for pollen and nectar (Balfour et al., 2018; Baude et al., 2016; Carvell et al., 2006; Goulson et al., 2002, 2005; Wignall et al., 2020).

Agriculture can also provide resources for pollinators. Mass‐flowering crops (MFCs) such as field bean (*Vicia faba*) and clover (*Trifolium* spp.) produce superabundant pollen and nectar while flowering (Bailes et al., 2015; Carruthers, 2016; Westphal et al., 2009). In the United Kingdom and other temperate countries, one of the most grown MFCs is oilseed rape (*Brassica napus*, OSR) which is used for vegetable oil, livestock feed and biofuel (Nedić et al., 2013). UK OSR increased from 390 thousand hectares cultivated in 1990 to 529 thousand hectares in 2019 (DEFRA, 2019) but declined to 364 thousand hectares in 2022 (DEFRA, 2023). Flowering OSR is visited by honeybees, bumblebees, solitary bees, and flies (Elzay & Baum, 2021; Jauker, Bondarenko, et al., 2012; Jauker, Peter, et al., 2012; Nedić et al., 2013; Westphal et al., 2009) and can enhance insect abundance and fitness during (Westphal et al., 2009) and after (Jauker, Peter, et al., 2012; Riedinger et al., 2015) bloom. Due to the reported benefits of OSR to insect fitness, some authors have suggested that further increasing nectar production via plant breeding programmes can further benefit insects by reducing floral resource shortages in otherwise resource‐poor agricultural landscapes (Carruthers, 2016; Carruthers et al., 2017; Fairhurst et al., 2021; Thom et al., 2016).

There has been recent interest in quantifying pollen and nectar production in OSR varieties (Carruthers et al., 2017; Fairhurst et al., 2021; Thom et al., 2016). However, no study has quantified demand for OSR floral resources by the insect community. Although floral resource supply in Britain has declined, whether resource supply meets demand varies between seasons due to flower phenology and changes in insect activity (Balfour et al., 2018; Timberlake et al., 2019). Spring (March–May) and autumn (September–November) have greater per capita floral resource supply than summer (June–August; Couvillon, Schürch, & Ratnieks, 2014; Timberlake et al., 2019; Wignall et al., 2020) due to declines of summer‐flowering plants resulting from historical land‐use change (Balfour et al., 2018). In the UK, most (95%) OSR blooms in spring (DEFRA, 2020), which has been suggested to have sufficient or surplus floral resource supply (Couvillon, Schürch, & Ratnieks, 2014; Timberlake et al., 2019). During the periods of low competition, a high percentage (>50%) of pollen and nectar produced by flowering plants can be left uncollected as there are too few insects to collect the resources produced (Harris et al., 2023). Monocultures of spring blooming MFCs might therefore contribute to an oversupply of floral resources in spring (Timberlake et al., 2019) and floral resources may be wasted by the flower‐visiting insect community. Honeybee waggle dances and pollen analysis in spring indicate limited foraging on nearby OSR fields and zero foraging on OSR greater than 2 km from the hives, suggesting that distant OSR fields cannot be profitably exploited relative to alternative food sources (Garbuzov et al., 2015). Moreover, Nedić et al. (2013) reported that honey yield produced by 40 honeybee colonies adjacent to a ten hectare OSR field was <10% of the estimated maximum, indicating that OSR was underutilised. If a high percentage of pollen and nectar produced by spring blooming OSR in Britain is uncollected, suggestions to produce cultivars of OSR which produce greater volumes of floral resources may be unneeded as additional resources will be wasted.

Although OSR and other MFCs have been shown to produce abundant nectar and pollen (Carruthers, 2016; Carruthers et al., 2017; Thom et al., 2016), floral resource production in relation to demand by flower‐visiting insects has not yet been examined. To better understand the use and possible wastage of floral resources from MFCs, we estimated the percentage of nectar and pollen produced by four spring blooming OSR fields per year over 2 years in Sussex, southeast England, which were uncollected by the flower‐visiting insect community. We also used estimates of nectar production, flower density and insect density to calculate estimated field‐scale nectar supply and nectar supply per insect. Additionally, to examine whether UK MFCs flower during months of greater or reduced competition, we used published datasets to estimate the area of UK MFCs in bloom per month.

## 2 MATERIALS AND METHODS

### 2.1 Study sites

Data were collected at four spring blooming OSR fields per year in mixed farmland in East Sussex, UK in 2021 and 2023. OSR fields were 5 to 98 hectares (mean, SD: 30 ± 30 ha; Table S1) and were at least 2 km apart. One field was surveyed during each study day. Data were collected on 12 study days in 2021 (between 15 April and 31 May) and eight study days in 2023 (9 April to 16 May) on days with suitable weather for insect foraging (low wind speed, >12°C). Vapour pressure deficit (VPD), which can influence the water content of nectar, was calculated for each study day following Fairhurst et al. (2021) using hourly relative humidity and temperature data for the study area provided by Met Office, UK (Crown Copyright, 2024; Table S1). Land use surrounding the study fields was mixed, consisting of arable fields, calcareous grassland, improved grassland, deciduous woodland, and urban/suburban areas.

### 2.2 Study species, *Brassica napus*

**FIGURE 1:**
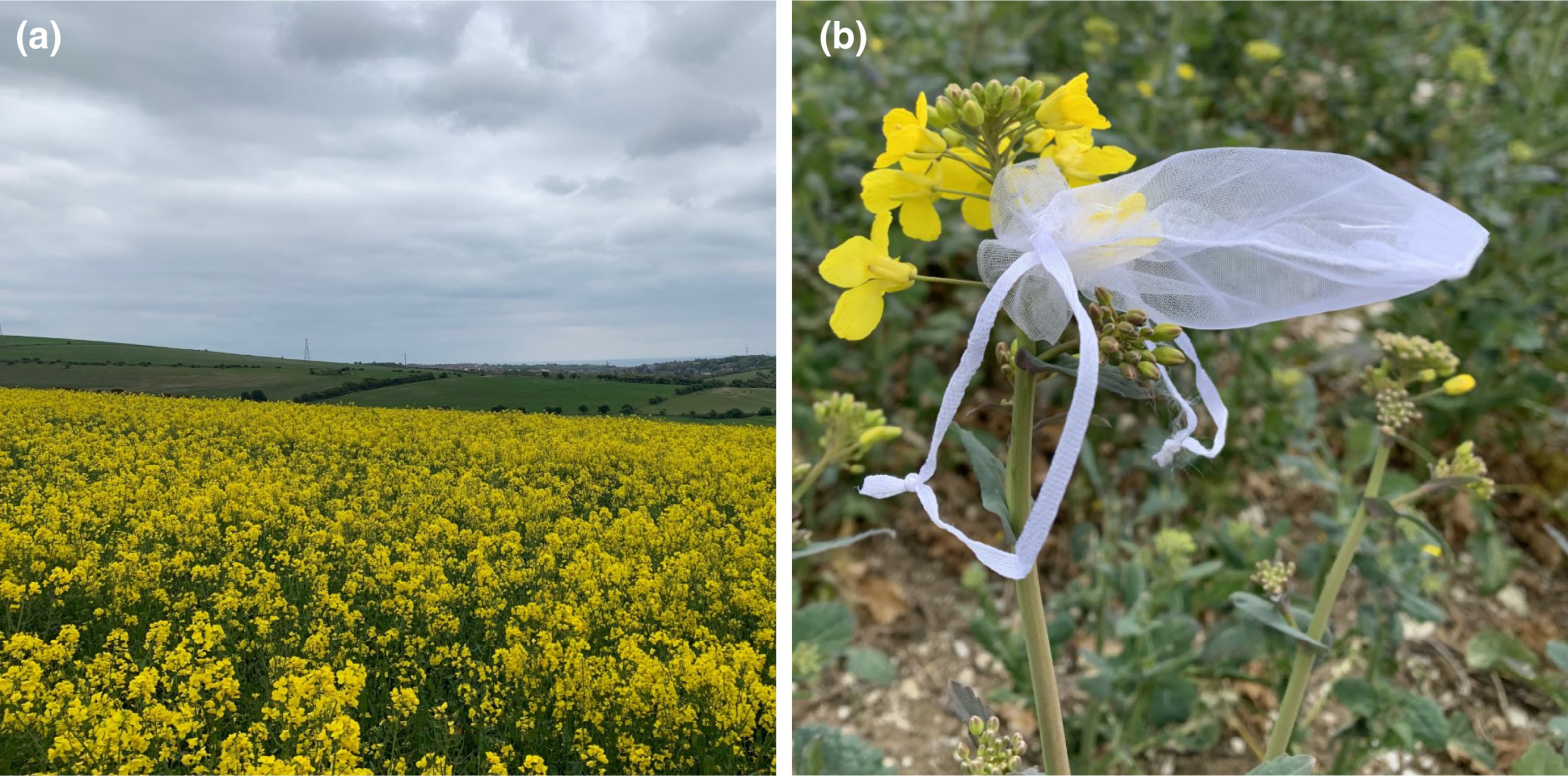
Oilseed rape (*Brassica napus*) (a) field in full bloom and (b) plant with a flower covered with a fine gauze bag to prevent insect access.

*Brassica napus* plants are usually sown at a density of 25–40 plants m^−2^ (AHDB, 2020), producing fields of dense flowers while in bloom (Figure 1a). Mature plants grow to 100–160 cm and produce flowers on racemes with the lowest buds flowering earliest (AHDB, 2020). Flowers typically last 1 to 2 days before wilting (Burquez & Corbet, 1991). Flowers have four radially symmetrical and horizontal petals that are apically widened providing a landing platform for foraging insects and allowing easy access to the nectaries (Nedić et al., 2013). OSR has anthers on six stamens, four positioned above the corolla and two within a cup‐like structure formed by the petals (Nedić et al., 2013). Anthers above the corolla protrude slightly allowing pollen‐collecting insects access to presented pollen (Jacobs et al., 2009, 2010). Wastage of pollen and nectar should not, therefore, be due to inaccessibility.

To confirm that OSR nectar is accessible to insects, residual nectar was quantified by inserting a 1 μL micropipette into the nectaries immediately (<5 s) after an insect foraged from a flower. Most, 62%, of samples had no detectable nectar and flowers that contained residual nectar contained minute quantities (mean residual nectar volume = 0.01 μL; *n* = 34 total residual nectar samples, honeybees *n* = 13, *Bombus* spp. *n* = 18, *Andrena* spp. *n* = 3).

### 2.3 Estimating percentage of nectar uncollected in individual flowers

Nectar can be defined as ‘wasted’ if it is left unconsumed by the flower‐visiting insect community, which will result in it being reabsorbed by the plant or discarded with the wilted flowers (Burquez & Corbet, 1991; Gill, 1978; Harris et al., 2023). To directly quantify the volume of nectar wasted, nectar accumulation was quantified and compared in flowers which were either accessible or inaccessible to insects. Flowers were made inaccessible by covering with fine gauze bags (manufacturer: Yoursfs, aperture size = 0.4 mm; Figure 1b). Gauze bags are recommended for measuring nectar production rate because it has minimal effect on the contained microclimate and therefore has little effect on nectar production (Corbet, 2003). Greater demand by the flower‐visiting insect community and more frequent insect visitation will cause lower nectar accumulation in accessible versus inaccessible flowers (Gill, 1978; Harris et al., 2023; Page & Williams, 2023).

Between 08:00 and 10:00, 10 pairs of flowers in the field were drained using 1 μL micropipettes. Flowers within a pair were on separate racemes on the same plant. One flower per pair was randomly selected to be inaccessible. To minimise the potential age differences, the highest open flower on each raceme was selected and both flowers were visually selected to be at a similar stage of development.

Five pairs were located along transects <10 m from the margin of the field and five along transects located centrally, >100 m, along tramlines. Pairs were > 20 m apart. The transects were the same used for insect surveys (see below).

Nectar samples were taken up to three times per study day (study periods T1: 09:00–12:00, T2: 12:00–15:00, T3: 15:00–18:00) 2–3 h apart (mean ± SD: 142 ± 26 min between readings). Nectar present in a flower was collected by inserting a 1 μL capillary tube (Drummond Microcaps) into the inner nectaries, and gently moved until all nectar was drained. Only the inner nectaries were sampled because the outer nectaries secrete little nectar, 5% (Davis et al., 1994). Nectar volume was determined by measuring the length of the nectar in the microcapillary tube using a ruler and calculating the percentage of the overall tube volume (Corbet, 2003). Nectar sugar concentration (% Brix) was determined by ejecting the nectar onto a handheld refractometer (Bellingham and Stanley™, 0–100% Brix). Nectar sugar mass (g) was calculated for each nectar sample by converting % Brix into mass/volume (Weast, 1972) and multiplying by volume (Corbet, 2003). Sugar mass/volume provided by Weast (1972) is provided in 0.5%, 1%, or 2% increments and therefore required interpolation. However, the error induced by this is minimal (Bolten et al., 1979).

Intervals between draining the flowers and sampling accumulated nectar was <3 h, which is insufficient time for nectar reabsorption to occur (Burquez & Corbet, 1991), and therefore represents the maximum potential supply. The actual amount of nectar available to the flower‐visiting insect community may be less than this as OSR flowers begin reabsorbing nectar solutes as they accumulate (Burquez & Corbet, 1991). Reabsorbed nectar sugar is wasted from the perspective of the insects, even if it is not wasted from the perspective of the plant.

### 2.4 Estimating percentage of pollen uncollected by insect community

To quantify pollen wastage, anthers were collected from the same plants that were used to collect the nectar samples. OSR flowers were shaken so that abscising anthers fell onto a piece of paper and transferred to Eppendorf tubes. As these anthers were close to abscission and would no longer accessible to insects, pollen in these anthers was therefore wasted from the perspectives of both insects and plants. Only anthers above the corolla were collected for analysis to ensure residual pollen was accessible. To collect fresh anthers, containing the maximum amount of pollen potentially available to insects, flowers at the beginning of anthesis were collected using forceps and stored in Eppendorf tubes. All anthers were stored at −14°C.

In the laboratory, one anther per patch per treatment was placed in a 2 mL Eppendorf tube with 15 μL 70% ethanol, sonicated at 40 khz for 3 min (RS PRO ultrasonic cleaner) and then vortexed for 30 s at 2000 rpm. 10 μL of liquid was then removed and placed on a haemocytometer (HIRSCHMANN®) and the number of pollen grains in each of the four corner squares was quantified using a microscope at 10x magnification. The difference between the two treatments (beginning versus end of anthesis) allowed estimation of the percentage of pollen wasted.

### 2.5 Quantifying insect and flower density

During each study period, ten 50 m transects were made by a recorder who walked along the transect and counted and identified insects actively visiting OSR flowers within 1 m to one side. Each transect walk lasted approximately 5 min. Five were carried out at the edge of the field (<10 m from the edge of the field) and five in the centre (>100 m) along tramlines, using the same transects used for nectar sampling. Flower‐visiting insects were identified to species where possible using field guides (Brock, 2021; Falk, 2018). Due to difficulty distinguishing *Bombus terrestris* and *Bombus lucorum* they were grouped into *Bombus terrestris* agg. Any insects which could not be identified in the field were caught and identified in the lab.

To quantify flower density, approximately 30 1 m^2^ quadrats (mean 31 ± 12 quadrats per study day) were placed every 20 m along a the same transects used to quantify insect density. Half were along the margins and half centrally. Numbers of racemes were counted in each quadrat. Additionally, approximately 20 racemes were randomly selected per transect and the numbers of open flowers were counted. The mean flower density per m^2^ was calculated for each study day by multiplying the mean number of racemes by the mean number of flowers per raceme.

### 2.6 Estimating field‐scale nectar supply

To calculate nectar supply per insect, mean flower density per m^2^, mean nectar secretion rate per flower (μL per hour of inaccessible flowers), and mean insect abundance per m^2^ were calculated for each study day. Nectar supply per m^2^ per hour was calculated for each study day as the product of mean flower density per m^2^ and mean nectar secretion rate per inaccessible flower. Total nectar supply per insect was calculated as the total nectar secretion per hour per m^2^ divided by mean insect density per m^2^, giving nectar production per insect per hour. Nectar sugar supply per insect was also calculated by repeating the above calculation with estimated nectar sugar secretion rate per flower (μg per hour of inaccessible flowers).

Because nectar was measured during short intervals (<3 h), nectar reabsorption was minimal. Therefore, these estimates are the potential maximum nectar supply.

### 2.7 Estimating field‐scale uncollected nectar

Overall nectar wastage was calculated for each study day by multiplying the mean nectar sugar accumulation from the accessible treatment by the mean flower density. To obtain an ecologically relevant metric for nectar supply and wastage, we converted estimated uncollected nectar sugar per hour into bumblebee colony equivalents. This was the estimated mean sugar uncollected per unit area per hour as a fraction of the total lifetime colony sugar intake of a *B. terrestris* colony, 1186 g (Rotheray et al., 2017).

### 2.8 Flowering phenology of UK mass‐flowering crops

Total area planted of UK oilseed rape (*Brassica napus*), field bean (*Vicia faba*), field pea (*Pisum sativum*), flax (*Linum usitatissimum*), orchard fruit (apples, *Malus* spp., and pears, *Pyrus* spp.), and soft fruit (strawberry *Fragaria* spp. and raspberry *Rubus* spp.) was obtained from DEFRA (2023). Beginning and end flowering dates were obtained from Baude et al. (2016). Total area planted was divided by the number of months each crop is in bloom; therefore, the area of crop in bloom was assumed to be constant throughout the flowering period of each crop. This gave an estimate of the total area of flowering MFCs per month.

### 2.9 Statistical analysis

Generalised linear mixed models using the R package glmmTMB (Brooks et al., 2017) were used. Model assumptions were tested and confirmed using the DHARMa package (Hartig & Lohse, 2020). Site nested within year were included as random factors for all models.

To assess how flower accessibility affected nectar sugar accumulation, treatment (accessible versus inaccessible) and within‐field location (edge versus inside) were fixed factors, and nectar sugar accumulation rate the response variable. To meet model assumptions, for each study day mean nectar secretion rate for each treatment was calculated per period (morning, midday, afternoon) and per location (edge versus inside). The model had a gaussian distribution.

To assess how anther age affected pollen count, treatment (beginning versus end of anthesis), within‐field location and their interaction were included as fixed factors with pollen count as the response variable. The model had a negative binomial distribution.

To examine how location in the field affected insect abundance, location (edge versus inside) was included as a fixed factor and insect abundance as the response variable. The model had a quasi‐Poisson distribution.

Models were simplified through stepwise removal of non‐significant variables and likelihood ratio tests provided p values for explanatory variables. Estimated marginal means were calculated using the R package emmeans (Lenth et al., 2024). All data analyses were conducted in R studio (R version 4.0.3; R Core Development Team, 2018).

## 3 RESULTS

**FIGURE 2:**
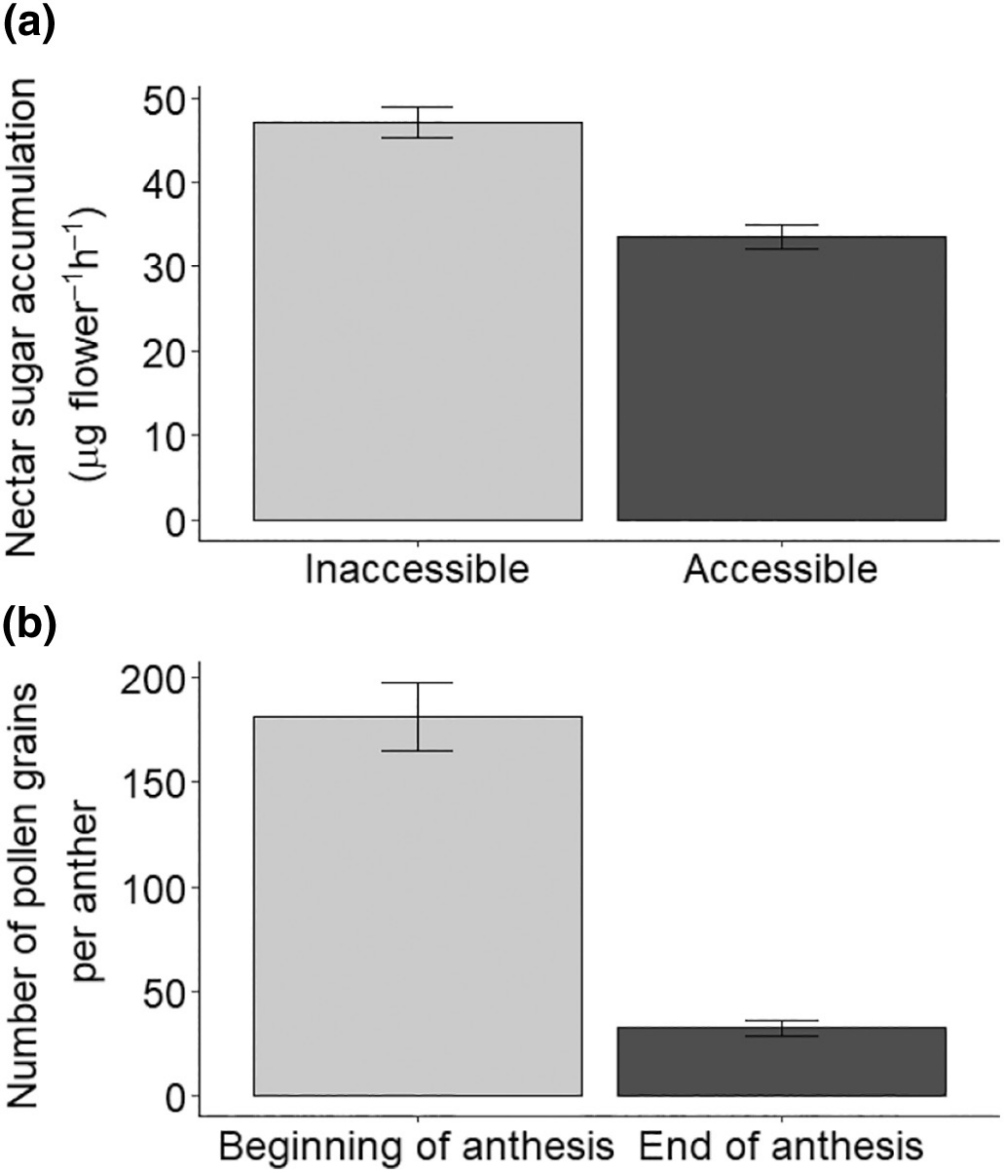
(a) Nectar sugar accumulation (mean ± SD) for OSR flowers which were inaccessible (*n* = 190) to flower‐visiting insects or accessible (*n* = 191; GLMM: χ^2^ = 37.1, df = 1, *p* < .001) and (b) haemocytometer pollen counts (mean ± SE) of anthers at the start (*n* = 116) and end (*n* = 147) of anthesis (GLMM: χ^2^ = 159.1, df = 1, *p* < .001). To measure nectar sugar accumulation, flowers were drained when weather became favourable to flower‐visiting insects and nectar was measured approximately every 3 h. Pollen counts were made from 10 μL from a suspension of one anther per patch in 15 μL of 70% ethanol. Data were collected on four OSR fields per year over 2 years (15 April to 31 May 2021 and 9 April to 16 May 2023).

93% of bagged and 96% of unbagged flowers contained measurable nectar when sampled. VPD during data collection was 0.48 ± 0.22 and 0.48 ± 0.29 for 2021 and 2023 respectively (mean, SD). Spring blooming OSR nectar accumulation in inaccessible (*n* = 190) and accessible (*n* = 191) flowers was 0.10 ± 0.08 and 0.07 ± 0.06 μL flower^−1^ h^−1^ respectively (mean, SD). Mean nectar concentration in inaccessible and accessible flowers was 42 ± 15 and 41 ± 17% (Brix) respectively (mean, SD). Nectar sugar accumulation in inaccessible and accessible flowers was 47.1 ± 39.5 and 33.6 ± 30.6 μg flower^−1^ h^−1^ respectively (mean, SD; Figure 2a).

Nectar sugar accumulation was significantly greater in inaccessible versus accessible flowers (χ^2^ = 37.1, df = 1, *p* < .001; Figure 2a). Estimated marginal mean nectar sugar in accessible flowers was 69% that of inaccessible flowers, indicating that 69% of nectar produced was uncollected by flower‐visiting insects (EMM ± SE, inaccessible: 40.8 ± 8.7, accessible: 28.2 ± 7.22). Nectar sugar accumulation was not significantly affected by within‐field location (χ^2^ = 3.6, df = 1, *p* = .06) or its interaction with treatment (χ^2^ = 0.10, df = 1, *p* = .75).

Pollen count per anther was significantly greater on anthers at the beginning of anthesis (χ^2^ = 159.1, df = 1, *p* < .001; *n* = 263 anthers; Figure 2b). Estimated marginal mean pollen count at the end of anthesis was 19% that of flowers at the beginning of anthesis (EMM ± SE, beginning: 167 ± 51.0, end: 32 ± 9.7), indicating that approximately 19% remained uncollected on the anthers. Pollen count was not significantly affected by within‐field location (χ^2^ = 0.8, df = 1, *p* = .4) or its interaction with treatment (χ^2^ = 0.3, df = 1, *p* = .57).

638 flower‐visiting insects were recorded. FVI density was 1.1 ± 1.6 insects per 50 m transect (mean, SD; *n* = 581 transects). Honeybees and bumblebees dominated, comprising 90% of observed insects (*Apis meliffera* 71%, *Bombus* sp. 19%, Table 1). Other insects included other bees 3%, Diptera 6%, and Lepidoptera 0.3% (Table 1).

**TABLE 1:** Insect species observed foraging on oilseed rape (*n* = 581 50 m^2^ transects).

Insect species or group	Family	Number observed
*Apis mellifera*	Apidae	454 (71%)
*Bombus terrestris* agg.	Apidae	92
*Bombus lapidarius*	Apidae	26
*Bombus pascuorum*	Apidae	4
**All bumble bees**		122 (19%)
*Andrena tibialis*	Andrenidae	4
*Andrena fulvago*	Andrenidae	3
*Andrena cineraria*	Andrenidae	3
*Andrena haemorrhoa*	Andrenidae	1
*Andrena scotica*	Andrenidae	1
*Andrena* spp.	Andrenidae	8
Halictidae	Halictidae	2
**All non‐apid bees**		22 (3%)
*Dasysyrphus venustas*	Syrphidae	2
Other Syrphidae	Syrphidae	4
Bombylius (bee fly)	Bombyliidae	2
Other Diptera		30
Vanessa cardui	Nymphalidae	1
Aglais io	Nymphalidae	1
**All non‐bees**		40 (6%)
**Total insects**		638

**FIGURE 3:**
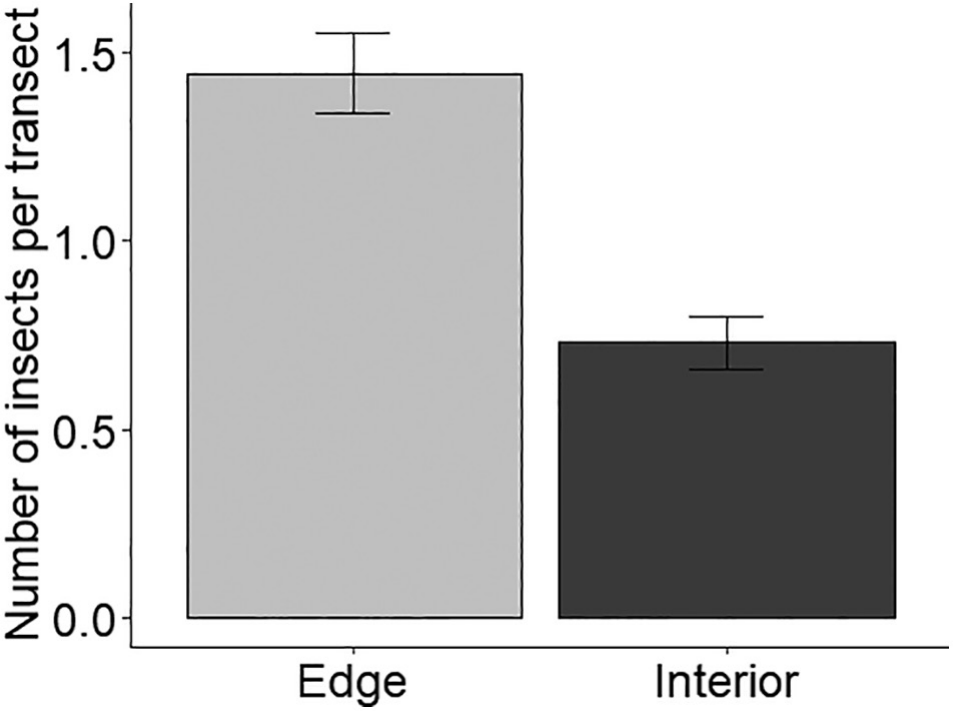
Insect abundance (mean ± SE) for 50 m transects walked at the OSR field edges (<10 m from field edge, *n* = 300 transects) and field interior (>100 m, *n* = 281; χ^2^ = 32.1, df = 1, *p* < .001). Data were collected on four OSR fields per year over 2 years (15 April to 31 May 2021 and 9 April to 16 May 2023).

Insect abundance was significantly associated with within‐field location (χ^2^ = 32.1, df = 1, *p* < .001; Figure 3), with estimated marginal mean insect abundance approximately two‐fold greater at the field edges than field interior (EMM ± SE, edge: 1.31 ± 0.71; interior: 0.71 ± 0.11).

Mean raceme density was 46 ± 25 m^−2^ (mean, SD; *n* = 608 quadrats) and mean flowers per raceme was 7 ± 5 flowers raceme^−1^ (*n* = 485 counts). Field‐scale OSR nectar sugar production was estimated to be 0.14 kg ha^−1^ h^−1^, or 0.12 bumblebee colony equivalents ha^−1^ h^−1^ across all study days. The accessible flowers indicated that 0.11 kg nectar sugar ha^−1^ h^−1^ was left uncollected by the flower‐visiting insect community, which equates to 0.09 bumblebee colony‐equivalents of nectar sugar ha^−1^ h^−1^. Mean OSR nectar supply per insect was estimated at 2204 μL nectar insect^−1^ h^−1^, equivalent to 1.1 g nectar sugar insect^−1^ h^−1^ (Table S1).

**FIGURE 4:**
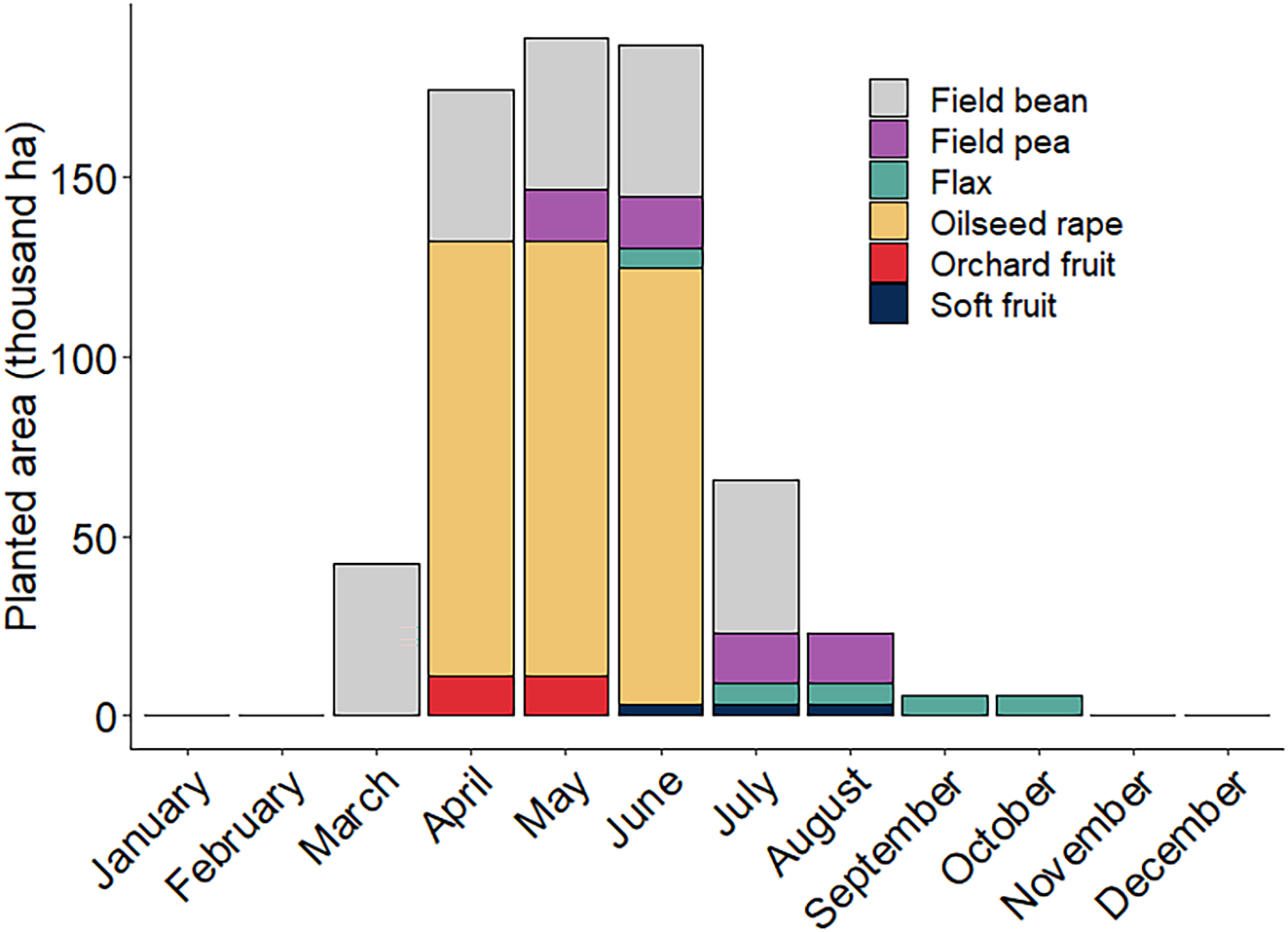
Flowering period of mass‐flowering crops and total area (ha) planted in the UK in 2022. Area planted for each crop was divided by the number of months in bloom. Orchard fruit includes apples (*Malus* spp.) and pears (*Pyrus* spp.) and soft fruit includes raspberries (*Rubus* spp.) and strawberry (*Fragaria* spp.). Phenology data from Baude et al. (2016) and area data from DEFRA (2023).

May was estimated to have the greatest area of flowering MFCs (230 thousand hectares), including oilseed rape, field bean, field pea, and orchard fruit (apples and pears; Figure 4). April and June also had high abundance of MFCs at 174 and 186 thousand hectares respectively (Figure 4). When taken together, spring (March–May) had the greatest area of flowering MFCs (406 thousand hectares), followed by summer (June–August; 276 thousand hectares), autumn (September–November; 12 thousand hectares), and winter (December–February; no flowering MFCs).

## 4 DISCUSSION

Our results show that that a high percentage, 69%, of nectar produced by OSR is wasted (Figure 2a). Additionally, spring (March–May) was estimated to have the greatest area of flowering MFCs (Figure 4), which is suggested to have lower exploitative competition (Couvillon, Schürch, & Ratnieks, 2014; Wignall et al., 2020) due to reduced insect activity and greater floral resource abundance (Balfour et al., 2018). This has important implications for the value of OSR and other MFCs as food sources for flower‐visiting insects and their role in conservation programmes. In particular, insects foraging on spring flowering OSR fields may not be limited by nectar supply and oversupply may occur. Although this would not be harmful to the insects, it does indicate there may be little conservation benefit in planting more spring flowering MFCs, especially in large fields, or of breeding for increased nectar production.

Over two thirds of the nectar, 69%, produced by the spring blooming OSR was uncollected in individual flowers. Additionally, field‐scale nectar supply was estimated to be 0.14 kg ha^−1^ h^−1^, or 0.12 bumblebee colony equivalents ha^−1^ h^−1^. This greatly exceeds the estimated nectar production rate of alternative flowers in other farmland habitats. For example, modelled peak nectar production on four mixed farms without OSR in southern England was estimated at 0.003 kg sugar ha^−1^ h^−1^ during spring (early April to late May; Timberlake et al., 2019). Moreover, even on farms without OSR, modelled spring nectar production was estimated to consistently exceed modelled bumblebee nectar demand (Timberlake et al., 2019).

Limited resource acquisition rate may also limit fitness without necessarily leading to complete resource depletion (Sponsler et al., 2023). However, estimates of per capita nectar supply in spring blooming OSR fields also indicate a nectar surplus. Based on our estimates of flower density, nectar production rate per flower and insect abundance, OSR produced a potential 2204 μL nectar insect^−1^ h^−1^, which equates to 1.1 g nectar sugar insect^−1^ h^−1^. Honeybees gather at least 15–20 μL nectar per foraging trip (Eckert et al., 1994), and *B. terrestris* workers can collect an average of 105 μL nectar per trip (Pattrick et al., 2020). Therefore, the estimated potential per capita nectar production rate exceeds the volumes collected on an average foraging trip by honeybees and *B. terrestris*, the two most frequent visitors to OSR fields, by a factor of approximately 100 and 20 respectively. Based on these estimates, the honeybees would need to make more than 100 foraging trips per hour to collect the nectar produced, which is clearly impossible. Moreover, we estimate that approximately 10% of the lifetime nectar requirement of a *B. terrestris* colony remains uncollected ha^−1^ h^−1^ in spring blooming OSR.

A lower percentage of pollen, 19%, was wasted, suggesting that OSR pollen may be used to a greater extent than nectar by flower‐visiting insects in spring. Insects can rapidly remove pollen from flowers. For example, on *Knautia arvensis* bumblebees and non‐*Bombus* bees can remove a high percentage (64% and 53% respectively on average) of presented pollen in a single visit (Larsson, 2005). Honeybees and bumblebees, the most frequently recorded groups on OSR, have high pollen requirements and OSR is an important pollen source for early colony growth (Cane & Tepedino, 2017; Westphal et al., 2009). Solitary bees, although less frequent, also have high pollen requirements, potentially requiring thousands of flowers to provision a single larva (Müller et al., 2006). Indeed, in some plant communities pollen availability can be consistently low even at lower levels of competition (Page & Williams, 2023). OSR is also partially wind pollinated (Hudewenz et al., 2014), and substantial amounts of pollen can be released by wind (McCartney & Lacey, 1991). However, any wind dispersal of pollen appears not to have eliminated pollen oversupply.

Our results show that a high percentage of nectar produced by spring blooming OSR is uncollected by flower‐visiting insects and therefore wasted. During spring, there are fewer insects on the wing (Balfour et al., 2018) and honeybee colonies have yet to reach maximum brood rearing and adult population size (Horn et al., 2021). Therefore, the large quantity of uncollected nectar in our study indicates that there may simply not be enough foraging insects to collect the superabundant resources produced by OSR (Elliott, 2009; Nicholls & Altieri, 2013). Indeed, insect density was consistently low at our study sites (mean 1.1 insects per 50 m transect). As a result, seemingly helpful suggestions to breed cultivars of OSR that produce greater quantities of nectar may be unneeded (Carruthers, 2016; Prasifka et al., 2018) as it would increase the surplus. Additionally, other UK MFCs primarily flower in spring (Figure 2a), indicating that most MFC floral resources are available during months of lower competition, where nectar supply is estimated to exceed demand even in the absence of MFCs (Timberlake et al., 2019).

Due to the prevalence of agricultural land, constituting approximately 70% and 38% of UK and EU land area respectively (DEFRA, 2023; Eurostat, 2022), incentivising farming practices that improve resource supply for insects during months or seasons that are more challenging for insect foraging is important. For example, summer blooming (spring planted) OSR, which flowers in summer where temporal gaps in nectar supply are more likely to occur (Couvillon, Fensome, et al., 2014; Couvillon, Schürch, & Ratnieks, 2014; Timberlake et al., 2019), may be more useful to insects by flowering when there is relatively less nectar, and probably pollen (Timberlake et al., 2019). Timberlake et al. (2019) estimated that nectar sugar supply fell short of modelled bumblebee sugar demand in late summer by an average of 10.53 g sugar ha^−1^ day^−1^. Nectar production by OSR would therefore greatly exceed this nectar shortage.

More broadly, the level of floral resource wastage in MFCs will likely be affected by the spatial distribution of OSR in the landscape. Central‐place foragers, which are ‘tied’ to habitats containing nest sites, will be less likely to be within foraging range of OSR if it is concentrated in large fields with large distances between them (Schürch et al., 2015). Alternatively, if the same area of OSR was distributed in more frequent but smaller fields a greater number of insects will be within foraging range. Models have shown that the proportion of honeybee colonies within foraging range of the nearest OSR field can vary greatly throughout Britain, between 6% and 79% of colonies in a region, due to the spatial distribution of OSR in the landscape (Schürch et al., 2015). Indeed, our data indicate that insect abundance was two‐fold greater at the field edge than inside (>100 m), suggesting that smaller fields will increase accessibility for insects and increase the percentage of available floral resources gathered (Woodcock et al., 2016). Examining resource wastage in OSR over a range of landscape contexts and field sizes will be a valuable area of further research for increasing resource accessibility for insects, particularly smaller‐bodied species which typically have smaller foraging ranges (Greenleaf et al., 2007; Woodcock et al., 2016), allowing insects to ‘make the most’ of resources produced by MFCs.

Local management practices will also likely affect resource wastage in MFCs. For example, in Britain honeybee colonies are frequently moved to OSR fields for pollination and honey production (Carreck et al., 1997), potentially increasing local insect density and resource demand (Page & Williams, 2023). The excess pollen and nectar produced by OSR indicates that moving honeybee hives would result in better use of the foraging resource provided. Indeed, moving honeybee hives to OSR fields could benefit other flower‐visiting insects by reducing competition for floral resources in the area from which they had been moved, allowing demand to follow supply.

Finally, potential trade‐offs between increased nectar production and other traits will be an important consideration. Excess nectar is not expected to be harmful to flower‐visiting insects but because nectar represents a significant energy investment in plants (Pleasants & Chaplin, 1983; Southwick, 1984), selection for increased nectar production may conflict with other functions such as seed production (Galetto et al., 2018; Pyke, 1991; Zimmerman & Pyke, 1988). Evidence suggests that agronomically beneficial traits such as early vigour, winter hardiness and stem strength are associated with nectar sugar and pollen quantity (Fairhurst et al., 2021), however further work is needed to determine whether pollen and nectar production influences crop yield or quality. Breeders should therefore aim for an optimum between supplying insects with sufficient resources and ensuring other agronomically beneficial traits are not negatively affected.

## 5 CONCLUSIONS

Our results show that a high percentage of nectar produced by OSR is not collected by flower‐visiting insects. Although OSR floral resource production has been quantified previously (Carruthers, 2016; Carruthers et al., 2017; Fairhurst et al., 2021; Thom et al., 2016), no study had measured this in relation to the demand by the insect community that use it. Our results demonstrate that although certain food sources or periods may be characterised by abundant floral resources, the value will also depend on the demand by the flower‐visiting insect community. For example, although Baude et al. (2016) reported that summer has the greatest nectar supply, most insects reach peak activity in summer (Balfour et al., 2018), resulting in low per‐capita nectar supply and greater exploitative competition (Timberlake et al., 2019; Wignall et al., 2020). Additionally, approximately half of pollen and nectar produced by the main autumn (September–November) food source in Britain, ivy (*Hedera helix*), are uncollected due to lower insect activity and high floral resource availability (Harris et al., 2023). This highlights that improving floral resources may not be as simple as ‘more is better’, particularly during periods, such as spring, where flower‐visiting insect numbers may be lower. Phenological and crop area data indicate that most UK MFCs flower during spring (March–May), indicating that resource oversupply may occur in other MFC species. Although we detected a surplus of nectar, a result that is qualitatively consistent with a lack of food limitation (Elliott, 2009), further work is needed to explore the fitness consequences of temporal gaps or surpluses in pollen and nectar supply, particularly acquiring a better understanding of the specific requirements of target insect species and how their needs change throughout their life cycle (Schellhorn et al., 2015). Moreover, because agricultural areas are often dominated by a small number of abundant plant species (Schellhorn et al., 2015), ensuring flower‐visiting insects also have access to sufficient quality and diversity of floral resources is important (Di Pasquale et al., 2013; Goulson et al., 2015; Vaudo et al., 2015). Our results suggest that a high percentage of floral resources produced by spring flowering mass‐flowering crops can be wasted by foraging insects, and conservation strategies should consider more than absolute resource supply.

## AUTHOR CONTRIBUTIONS

**Ciaran Harris:** Conceptualization (equal); data curation (lead); formal analysis (lead); investigation (equal); methodology (equal); resources (equal); software (equal); validation (equal); visualization (lead); writing – original draft (lead); writing – review and editing (equal). **Nicholas J. Balfour:** Data curation (supporting); investigation (supporting); methodology (supporting); writing – review and editing (supporting). **Francis L. W. Ratnieks:** Conceptualization (equal); data curation (supporting); formal analysis (supporting); funding acquisition (lead); methodology (equal); project administration (equal); resources (equal); supervision (equal); writing – original draft (supporting); writing – review and editing (equal).

## FUNDING INFORMATION

Funding for this research was provided by the Elizabeth Creak Charitable Trust who are funding CH's PhD.

## CONFLICT OF INTEREST STATEMENT

The authors declare that they have no financial/personal relationships that may be considered as potential competing interests.

## Supporting information


Table S1.

## Data Availability

The data that support the findings of this study will be openly available in Mendeley Data.
