# Supplementary material for: Floral resource wastage: Most nectar produced by the mass‐flowering crop oilseed rape (Brassica napus) is uncollected by flower‐visiting insects
Source: Ecol Evol. 2024 May 21;14(5):e11453. doi: 10.1002/ece3.11453 (PMC11106685; doi:10.1002/ece3.11453)
Supplement: Supplementary file 1 — Table S1. [file ECE3-14-e11453-s001.docx]

Table S1. Summary of estimates for each study day. Nectar production was calculated as the product of flower per m^2^ and nectar production per flower. Per capita nectar production was calculated by dividing the estimated sugar production per m^2^ by insect density.

| Date | OSR variety | Vapour Pressure Deficit (Kpa) | Site | Field size (ha) | Flowers per raceme | Racemes per m^2^ | Flowers per m^2^ | Nectar production rate (µl per flower per hour) | Nectar production (µl per m^2^ per hour) | Nectar concentration (µg sugar per µl) | Nectar sugar production (µg per m^2^ per hour) | Insect density (individuals per m^2^) | Per capita nectar volume (µl per insect per hour) | Per capita nectar sugar (g per insect per hour) |
| --- | --- | --- | --- | --- | --- | --- | --- | --- | --- | --- | --- | --- | --- | --- |
| 15/04/2021 | Inventive | 0.45 | A | 50 | 8.0 | 28.3 | 226.3 | 0.037 | 8.3 | 343.2 | 2514.4 | 0.006 | 1401.4 | 0.42 |
| 18/04/2021 | Aspire | 0.41 | B | 98 | 6.9 | 5.0 | 34.5 | 0.027 | 0.9 | 501.1 | 504.8 | 0.005 | 190.1 | 0.10 |
| 20/04/2021 | Barbados | 0.29 | C | 20 | 8.6 | 9.2 | 79.4 | 0.091 | 7.2 | 368.1 | 2381.1 | 0.028 | 260.9 | 0.09 |
| 23/04/2021 | Inventive | 0.80 | A | 50 | 11.3 | 28.9 | 325.0 | 0.051 | 16.6 | 536.8 | 8962.1 | 0.016 | 1058.7 | 0.57 |
| 26/04/2021 | Temptation | 0.62 | D | 16 | 8.2 | 68.2 | 557.3 | 0.054 | 29.9 | 534.6 | 15700.4 | 0.014 | 2172.2 | 1.14 |
| 30/04/2021 | Barbados | 0.51 | C | 20 | 9.2 | 29.6 | 273.6 | 0.053 | 14.5 | 481.8 | 6615.5 | 0.012 | 1172.0 | 0.53 |
| 02/05/2021 | Temptation | 0.31 | D | 16 | 6.9 | 64.5 | 445.3 | 0.072 | 32.1 | 341.4 | 9391.1 | 0.032 | 1003.0 | 0.29 |
| 07/05/2021 | Aspire | 0.57 | B | 98 | 7.7 | 87.4 | 672.9 | 0.056 | 37.9 | 567.8 | 21720.0 | 0.004 | 9469.6 | 5.43 |
| 11/05/2021 | Inventive | 0.29 | A | 50 | 6.7 | 101.0 | 678.7 | 0.146 | 99.1 | 479.4 | 48926.3 | 0.013 | 7429.1 | 3.67 |
| 14/05/2021 | Barbados | 0.31 | C | 20 | 6.0 | 55.6 | 335.2 | 0.135 | 45.3 | 328.8 | 15509.5 | 0.013 | 3487.9 | 1.19 |
| 25/05/2021 | Temptation | 0.49 | D | 16 | 1.0 | 30.0 | 30.0 | 0.097 | 2.9 | 521.6 | 1480.3 | 0.050 | 58.4 | 0.03 |
| 29/05/2021 | Inventive | 0.74 | A | 50 | 1.0 | 17.6 | 17.6 | 0.083 | 1.5 | 606.0 | 935.4 | 0.071 | 20.4 | 0.01 |
| 09/04/2023 | Aspire | 0.08 | E | 20 | 1.0 | 50.9 | 50.9 | 0.133 | 6.8 | 388.7 | 2524.4 | 0.017 | 407.0 | 0.15 |
| 17/04/2023 | Aspire | 0.26 | E | 20 | 9.2 | 33.2 | 305.9 | 0.188 | 57.6 | 425.6 | 25111.0 | 0.031 | 1837.9 | 0.80 |
| 19/04/2023 | Astrid | 0.61 | F | 5 | 9.0 | 24.2 | 217.8 | 0.124 | 27.1 | 530.0 | 15109.8 | 0.006 | 4517.2 | 2.52 |
| 30/04/2023 | Aspire | 0.30 | G | 13 | 7.8 | 46.9 | 365.4 | 0.174 | 63.4 | 557.8 | 35673.8 | 0.016 | 3963.5 | 2.23 |
| 03/05/2023 | N/A | 0.76 | H | 21 | 8.7 | 42.7 | 371.5 | 0.113 | 42.1 | 599.5 | 26789.3 | 0.037 | 1148.6 | 0.73 |
| 11/05/2023 | Astrid | 0.32 | F | 5 | 8.1 | 51.6 | 420.17 | 0.150 | 62.9 | 514.8 | 33243.5 | 0.034 | 1849.2 | 0.98 |
| 15/05/2023 | Aspire | 0.74 | G | 13 | 4.0 | 34.1 | 136.4 | 0.057 | 7.8 | 406.5 | 4181.1 | 0.004 | 2170.7 | 1.17 |
| 16/05/2023 | N/A | 0.78 | H | 21 | 5.2 | 41.9 | 215.8 | 0.068 | 14.6 | 457.9 | 7547.9 | 0.032 | 455.5 | 0.24 |
